# Non-invasive techniques for studying macrophages in joint inflammation

**DOI:** 10.1186/1471-2474-16-S1-S10

**Published:** 2015-12-01

**Authors:** Harrie Weinans

**Affiliations:** 1 UMC Utrecht, The Netherlands

## 

Folate-based radiotracers have been used in patients with cancer and inflammatory diseases to visualize folate receptor expressing cells using PET or SPECT techniques. Activated macrophages express folate receptor beta (FR-β) and this allows specific imaging of these cells *in-vivo*. From previous work using SPECT imaging to visualize folate receptor expressing macrophages in both animal models and in patients with OA we know that macrophages are present in OA affected joints. However, it remains unclear what role these macrophages play in the different stages of OA and whether their role can be influenced by specific targeting.

In Wistar rats osteoarthritis was induced using a low dose of intra-articular papain injections in one knee joint combined with exposure to a moderate exercise protocol. After six weeks and twelve an *in vivo* folate SPECT/CT scan and micro-CT analyses were performed. Macrophages from human peripheral blood monocytes were cultured (7 days) in the presence of GM-CSF (M1 proinflammatory phenotype) or M-CSF (M2 anti-inflammatory phenotype). Subsequently the macrophages were treated with LPS, cytokines (IL-4, IL-10, IFN-y) or a corticoid steroid (triamcinolone acetonide, 1µg/ml). Folate receptor beta (FRβ) as well as other macrophage marker expressions were measured using FACS.

Intra-articular injections with triamcinolone strongly enhanced FRβ+ macrophage activation and fully prevented osteophyte formation. There were no beneficial effects of the corticoid steroid against cartilage degradation or subchondral bone sclerosis. In in-vitro cultures triamcinolone strongly induced the monocyte-macrophages differentiation towards CD163+ and FRβ+ cells, specifically in GM-CSF stimulated (M1) cultures. Addition of triamcinolone to M-CSF stimulated (M2) monocytes showed enhanced IL10 expression on mRNA level.

In conclusion triamcinolone enhanced FRβ expression in monocytes that were induced to macrophage differentiation. The triamcinolone injections stimulate synovial macrophage activation and triggers the macrophages towards a more anti-inflammatory subtype.

**Figure 1:**
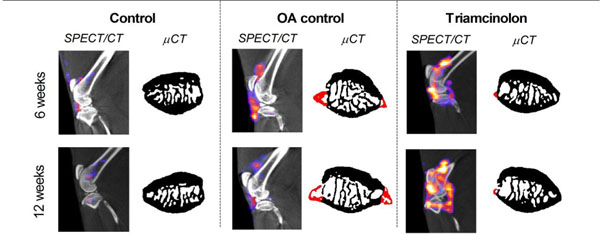
Macrophage activation determined after injection of 111In-DTPA-folate using SPECT/CT in Papain induced osteoarthritis (OA) combined with moderate exercise. Representative sagittal SPECT/CT images of knee joints from representative animals per experimental group. CT images shown in black and white were used for anatomical reference, the SPECT images are shown in colour. Patellar bone is shown with osteophyte formation highlighted in red.

